# Risk factors for intraocular penetration of caterpillar hair in Ophthalmia Nodosa: A retrospective analysis

**DOI:** 10.4103/0301-4738.71711

**Published:** 2010

**Authors:** Sabyasachi Sengupta, Padmati Ravindranath Reddy, Jamyang Gyatsho, Ravilla D Ravindran, Thiruvengadakrishnan, Vikram Vaidee

**Affiliations:** Department of Cornea and Refractive Services, Aravind Eye Hospital and Postgraduate Institute of Ophthalmology, Pondicherry, India

**Keywords:** Caterpillar hair, intraocular penetration, ophthalmitis

## Abstract

We report risk factors associated with intraocular penetration of caterpillar hair seen at our institute from January 2005 to December 2007. Records of all patients with caterpillar hair induced ophthalmitis (CHIO) were retrospectively reviewed for clinical characteristics, anatomic location of lodgment of the caterpillar hair, treatment methods, and outcomes. Out of a total of 544 cases of CHIO, 19 eyes (seven in the anterior chamber and 12 in the posterior segment) experienced intraocular penetration (3.5%). The presence of deep intracorneal hair (80 cases, 14.7%) was found to be the only risk factor for intraocular penetration (*P* < 0.001). The removal of intracorneal hair was possible in only 29 out of 80 eyes (36%) and this was associated with a significantly reduced risk of intraocular penetration (*P* = 0.022). Patients with retained intracorneal hairs should be counseled regarding risk of intraocular penetration and closely followed up for at least six months.

Caterpillar hair induced ophthalmitis (CHIO) is an inflammatory response of ocular tissue to caterpillar hair or other insect hair (tarantula hair) that come in contact with the eye. It is usually an innocuous condition and responds readily to conservative management. However, a small number of patients experience intraocular penetration of caterpillar hair, with potentially disastrous complications. Most existing literature on intraocular penetration of caterpillar hair is in the form of anecdotal case reports and small case series.[1–9] However, risk factors that may predict the occurrence of this event have not been elucidated. The basis of our study was to identify the risk factors that predict intraocular penetration of caterpillar hair.

## Materials and Methods

Medical records of all cases of CHIO diagnosed from January 2005 to December 2007 were drawn from a computerized data base. The demographic features, clinical features, medical and surgical treatments, and outcomes of the therapy were reviewed. All patients underwent slit-lamp examination and indirect ophthalmoscopy to determine the anatomical location as well as the number of caterpillar hair. Pediatric patients (one month to 10 years) were evaluated under general anesthesia (EUGA) and operating microscope if required. The clinical manifestations varied significantly and were classified into various types as suggested by Cadera *et al*.[9]

Type I: An acute, anaphylactoid reaction to the hair, starting immediately and lasting a few days, causing chemosis and inflammation.

Type II: Chronic mechanical kerato conjunctivitis caused by hair lodged in the bulbar or palpebral conjunctiva, leading to linear corneal abrasions.

Type III: Formation of grayish-yellow granulomatous nodules in the conjunctiva. The hair may be subconjunctival or intracorneal and may be asymptomatic.

Type IV: Iritis secondary to hair penetration into the anterior segment. The iritis may be very severe with iris nodule formation and even a hypopyon.

Type V: Vitreoretinal involvement after hair penetration into the posterior segment.

As a routine, on the first visit, an attempt to remove the visible superficial hair was made by the treating ophthalmologist. Medical therapy varied between lubricants and antibiotic eye ointments, with a bandage and additional topical steroids, when granulomatous inflammation (nodule) was observed. Systemic steroids were resorted to when posterior segment involvement was seen. Risk factors for intraocular penetration were analyzed using the Fischer’s exact test and Chi-square test.

## Results

**Figure 1:**
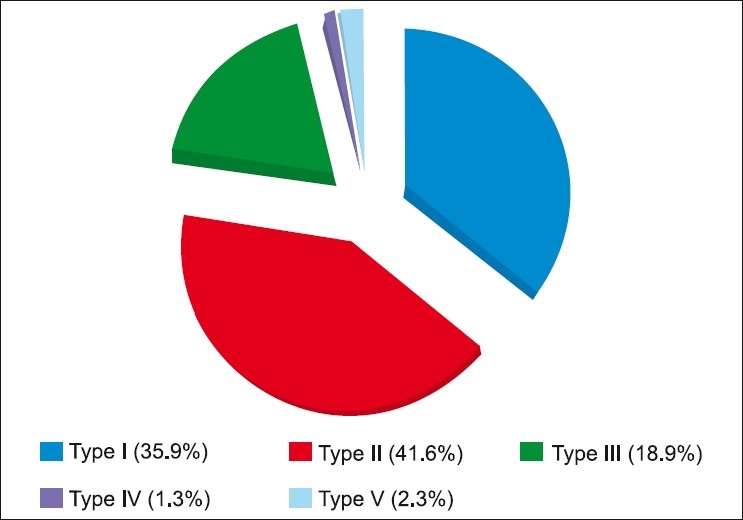
Distribution of patients according to the type of reaction

A total of 544 eyes with CHIO were identified over a three-year study period, out of which 19 eyes developed intraocular penetration (3.5%) of hair. The mean age was 33.64 years (Range from one month to 83 years).Fig. 1 shows the distribution of patients according to the type of reaction. Anatomical location of the lodgment of the caterpillar hair is shown in Table 1.

**Table 1:** Anatomical location of the lodgment of the hair

Location	Absolute number	Percentages
UTC	364	67.03
LTC	107	19.7
UTC + LTC	40	7.36
Cornea	79	14.54
Anterior chamber / Iris	7	1.28
Vitreous	4	0.73
Retina	9	1.65

UTC: Upper tarsal conjunctiva, LTC: Lower tarsal conjunctiva

**Figure 2:**
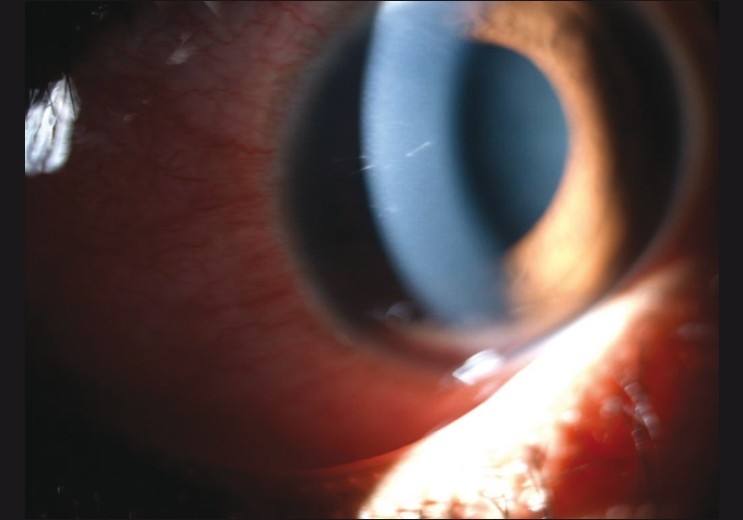
Retained intracorneal setae, tangential to the corneal curvature, with minimal surrounding congestion

Out of the 80 eyes with intracorneal hair at presentation, 19 eyes (23.75%) demonstrated intraocular migration. Presence of intracorneal hair was the only factor that was significantly associated with penetration of hairs (*P* < 0.001). Successful removal of the hair was possible in only 29 instances (36%), while in the remaining 51 eyes (64%), they were retained [Fig. 2]. Eyes with complete removal of the hair experienced significantly lesser intraocular penetration (6.9%) compared to those eyes in which the hair was retained (33.33%) (*P* = 0.02). Other risk factors considered for intraocular migration did not reach statistical significance [Table 2].

**Table 2:** Analysis of risk factors associated with intraocular penetration of caterpillar hair

Variable	Category	N	Intraocular Penetration	Percentage	*P* value
Age	Adult	490	17	3.47	0.99
	Pediatric	54	2	3.70	
Gender	Male	297	12	4.04	0.44
	Female	247	7	2.83	
Direct Contact with caterpillar	Yes	95	5	5.26	0.35
	No	449	14	3.12	
Eye Involved	Right eye	274	9	3.28	0.79
	Left Eye	270	10	3.70	
Duration of Symptoms	1 – 3 days	374	13	3.48	0.936	
	3 – 7 days	106	4	3.77	
	>7 days	64	2	3.13	
Total Number of hair on ocular surface	1 – 5 hair	280	8	2.86	0.42
	> 5 hair	264	11	4.17	
Presence of intracorneal hair	Yes	80	19	23.75	< 0.001
	No	464	0	0
Removal of intracorneal hair	Removed	29	2	6.90	0.022
	Retained	51	17	33.33	

**Figure 3:**
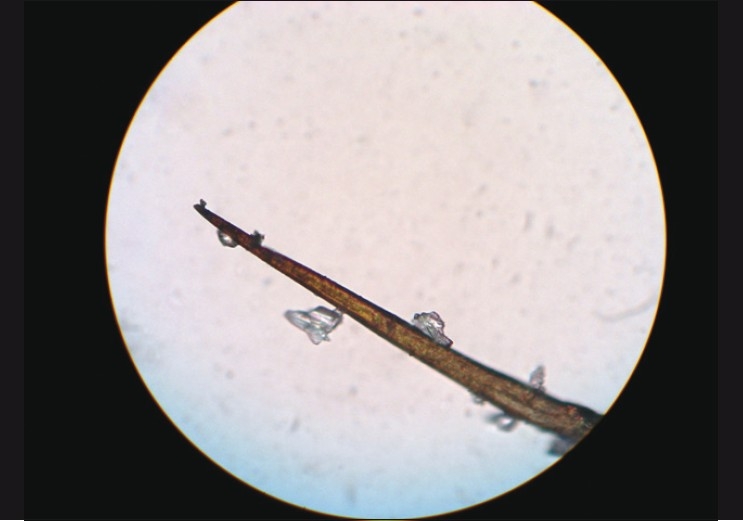
Caterpillar hair at 40× magnification showing spines along the shaft with epithelial debris adherent to the spines

**Figure 4:**
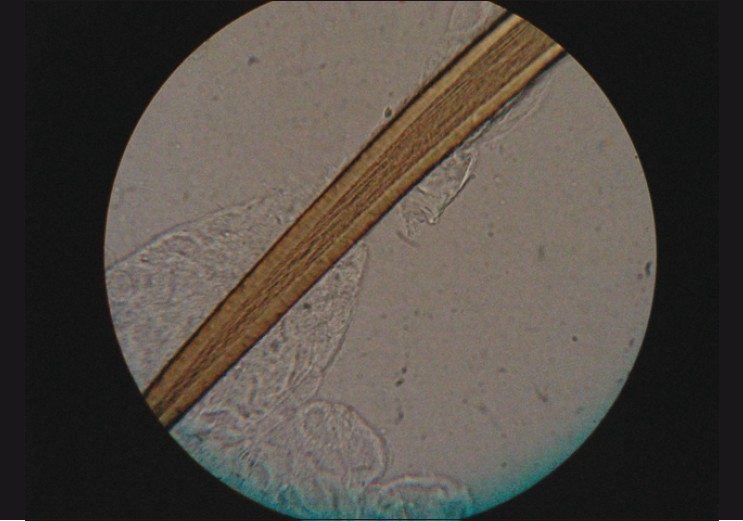
Caterpillar hair at 40× magnification with no spines along the shaft

Intraocular penetration of hair into the iris, vitreous, and retina were seen at variable intervals ranging from a few days to as long as six months from the initial presentation on the ocular surface. One patient with intravitreal hair developed persistent vitritis and received 4 mg/ 0.1 ml intravitreal triamcinolone acetonide (IVTA) injection to control inflammation at the three-month follow-up. Three patients with retinochoroiditis were well-controlled with a short course of oral steroids. One pediatric patient experienced endophthalmitis with retinal detachment within three weeks, finally resulting in phthisis bulbi. Hair isolated from the conjunctiva was studied randomly under the light microscope, at 40X magnification, in 12 of the cases. Two different varieties were seen, one with spines along the shaft [Fig. 3] and the other without spines [Fig. 4].

## Discussion

The prevalence of CHIO is high in this part of the world as evidenced by our large sample over a relatively short period of time. To the best of our knowledge, this is the first study evaluating the risk factors for intraocular penetration of caterpillar hair.

We found the presence of intracorneal hair to be the only significant risk factor for intraocular penetration, with potentially devastating complications. Successful removal of all intracorneal hair led to a significant reduction in the risk of intraocular penetration. However, it is very difficult to remove the hair in all instances due to their extreme friability, accompanying corneal edema, surrounding infiltration, and deep lying hair. Most patients have more than one hair, all of which may not be amenable for removal at the first sitting. Thus, patients with retained intracorneal hair must be followed up closely as vision-threatening complications may develop late in the course of the disease.

The pathological damage caused by a caterpillar hair is a function of its direct toxicity and locomotion. The force with which the hair strikes the eye may determine the risk of intraocular penetration. However, the quantum of hair present on the ocular surface and direct contact with a caterpillar do not influence the risk of penetration as seen from our analysis.

Frank endophthalmitis is very rare, although mild grades of vitritis have been more commonly reported. Posterior segment involvement may occur early or even years later. The majority of patients with vitreoretinal hair, in our series, did not have anterior chamber hair favoring transcleral penetration. Late onset endophthalmitis requiring vitrectomy has been reported despite adequate treatment of the anterior segment manifestations.[2,3]

In conclusion, a majority of the cases of CHIO are limited to manifestations of the type I to type III variety, which respond well to removal of the hair and standard topical steroid management. The prognosis is relatively good even with intraocular penetration of the hair. Despite the grave range of possibilities in the manifestations, the outcome in most of the cases is satisfactory, if diagnosed early and treated appropriately. The presence of intracorneal hair is a significant risk factor for intraocular penetration.
